# Nd:YAG Laser Vitreolysis for Symptomatic Vitreous Floaters: Application of Infrared Fundus Photography in Assessing the Treatment Efficacy

**DOI:** 10.1155/2019/8956952

**Published:** 2019-01-27

**Authors:** Xiaolei Sun, Jingyi Tian, Jinyan Wang, Jingjing Zhang, Ying Wang, Gongqiang Yuan

**Affiliations:** ^1^Medical College, Qingdao University, 308 Ningxia Road, Qingdao 266071, Shandong, China; ^2^Shandong Eye Hospital, Shandong Eye Institute, Shandong Academy of Medical Sciences, Jinan 250021, Shandong, China

## Abstract

**Background:**

Vitreous floater is a physically common phenomenon with aging and is related to visual impairment and decrease of quality of life. Nd:YAG vitreolysis is supposed to be an option for resolving floaters, but its clinical efficacy is undefined. We aimed to evaluate the efficacy of Nd:YAG vitreolysis in treating floater semiquantifiably by determining changes of floater areas on infrared fundus photography (IR).

**Methods:**

Patients with floaters and those who underwent Nd:YAG vitreolysis were retrospectively summarized from June 2015 to Nov 2017. Intraocular pressure, visual acuity, visual function questionnaire (VFQ-25) scores, and floater areas calculated using Image J software were recorded preoperatively and 6 months after YAG lasers.

**Results:**

50 patients (25 female/25 male, with an average age of 60.34 years) with 55 eyes (29 OD and 26 OS) presenting floaters and underwent YAG vitreolysis treatment were included. Severe symptoms were reported in 17 eyes, moderate in 21 and mild in 17 eyes. No severe Nd:YAG vitreolysis procedure-related complications occurred in all patients except one mild retinal injury. There were no significant changes in intraocular pressure and visual acuity after the laser treatment. 43 eyes had improved symptoms; in 8, floaters had disappeared; and 4 had no changes according to VFQ-25 scores. The median of shadow areas of floaters before operation was 1.41 (0.29–12.85) cm^2^, which decreased to 0.12 (0–2.77) cm^2^ after the operations (*t*=5.849, *P*=0.001). The mean VFQ-25 scores increased to 88.54 ± 12.74 from the baseline 71.44 ± 12.77 (*t*=11.82, *P*=0.001). Pearson correlation analysis showed that the shadow areas of floaters were negatively correlated to VFQ-25 scores before (*r*=−0.73, *P*=0.001) and after (*r*=−0.72, *P*=0.001) treatments.

**Conclusion:**

Nd:YAG vitreolysis was effective and safe in alleviating the visual symptoms induced by floaters. Quantification of floater shadow areas on infrared fundus photography could serve as an objective index for assessing treatment efficacy of Nd:YAG vitreolysis.

## 1. Introduction

The human vitreous body is an optically transparent gel-like structure within the eye consisting of approximately 98% water and macromolecules such as collagen and hyaluronic acid [1]. It is showed that the composition and organization of vitreous body change with aging, accompanied by the increased risk of posterior vitreous detachment (PVD). In addition, conditions such as myopia and diabetes may also exacerbate the vitreous liquefaction procedure and result in the formation of intravitreal collagen aggregates [2]. These intravitreal opacities may cast shadows onto the retina. The patients see them as black or grey structures moving in the visual field with appearance of line, dots, flies, or other shapes. Therefore, this phenomenon is clinically described as “vitreous floaters” [3].

Over the past decades, the symptom of vitreous floaters is routinely considered as a tolerable problem in most clinical settings, and their adverse impacts on patients' vision and quality of life are always underestimated [1]. Currently, more patients suffering from symptomatic vitreous floaters have clinically managed to get rid of the symptoms [4, 5]. As a potent noninvasive treatment modality for floaters, neodymium-doped yttrium aluminum garnet (Nd:YAG) laser vitreolysis is considered as a safe procedure with minimal complications [6]. Nd:YAG laser excites short, intense pulses and produces energy to vaporize the vitreous opacities to plasma by raising the reginal temperature to above 1000 Kelvin at a confined spot [7, 8]. However, there are limited studies focusing on the treatment efficacy of Nd:YAG on vitreous floaters currently. Moreover, the treatment efficacy of Nd:YAG is mainly defined by subjective self-reported improvement on visual symptoms, and less quantitative studies focusing on the appearance and area are available. Infrared (IR) imaging is another form of conventional fundus photography imaged at an observation wavelength of 820 nm. The use of infrared light makes it possible to detect details of the retina [9]. Our study aims to describe the treatment efficacy of Nd:YAG on vitreous floaters by evaluating the changes of floater areas in fundus infrared imaging.

## 2. Materials and Methods

### 2.1. Patients

Patients diagnosed of vitreous floaters under B-ultrasound and slit-lamp microscope and underwent Nd:YAG laser vitreolysis were retrospectively enrolled in this study in our hospital from June 2015 to November 2017. The patients resorted to Nd:YAG (Ultra Q Reflex-YAG, Ellex Medical, Australia) vitreolysis treatment for symptomatic floaters due to life disturbance. They had no systemic severe diseases and symptomatic progression of floaters within the past two months. The exclusion criteria were (1) floater/floaters located within 2 mm from the retina or the crystalline lens; (2) vitreous hemorrhage and other severe vitreous pathologic floaters; (3) a history of intravitreal injections or intraocular surgery; (4) complicated with vitreous proliferation, uveitis, fundus lesion, and other severe ocular diseases; (5) patients had risk of retinal detachment; and (6) patients were lost in the follow-up. This study was approved by the ethics committee of our hospital, and informed consents were obtained from all patients. This study adhered to the tenets of the Declaration of Helsinki. All patients were questioned with the duration of their symptoms prior to presentation, laterality, severity, and number of their floaters and activity most inconvenienced by the presence of floaters.

### 2.2. Surgical Procedure

All included patients performed visual acuity, intraocular pressure, B-ultrasound, and fundus examinations before YAG laser. YAG vitreolysis was performed using the Ultra Q Reflex laser by the same treating physician. The study eye was dilated with tropicamide eye drops preoperatively. The energy was initially set at 2.5–3.5 mJ and titrated to an appropriate level until plasma formation with the creation of gas bubbles. A maximum energy per pulse of 5.5 mJ was used. The number of laser shots given per patient was at the surgeon's discretion but no more than 500 accumulative pulses within 20 min in all cases. Laser administration was ceased after vaporization of the Weiss ring and all other visually significant floaters. If there were more residual floaters, another laser treatment was performed after 1 week.

### 2.3. Subjective Evaluation

The National Eye Institute Visual Functioning Questionnaire-25 (NEI VFQ-25) was completed by each patient before operation and 6 months after the last procedure. The subjective percentage of improvement was quantified by the patients and was recorded as follows: failure: floaters were the same or worse (0–30%); partial success: some improvement but still floaters of moderate inconvenience (31–70%); significant success: significant improvement with only slight inconvenience (71–99%); complete success: complete resolution of floaters (100%).

### 2.4. Infrared Fundus Photography and Objective Outcome

The infrared fundus images were obtained using the Heidelberg Retina Angiograph 2 system with scanning fields of 35° or 55° at a wavelength of 820 nm in each patient before laser administration and at 6 months follow-up. Ten images (8.8 frames/s) were averaged with the Automatic Real-Time composite mode of the instrument to obtain high-quality images. Grey-level analysis on a subset of images was performed using ImageJ software (version 1.43u, National Institutes of Health, Bethesda, MD, USA) to evaluate the distribution and areas of the floaters. All images (768 × 868 pixels) were imported into the Image J software and then were converted to 8-bit type files. After adjusting for certain brightness and contrast, the image scale was identified at a unit of centimeter (cm)/pixel. The target areas with floater shadow were circled on the images and measured automatically by the software from at least 5 independent images for each patient. The floaters areas were analyzed by two physicians in this study except the surgeon. The change of floater areas was calculated, and the objective efficacy was defined as follows: failure: 0–30%; partial success: 30–70%; significant success: 70–99%; and complete success with complete disappear of floater shadow.

### 2.5. Statistical Analysis

Continuous variables were presented as means ± SD in normally distributed variables or median with minimum to maximum in skewed variables and were compared using a paired *t*-test or McNemar nonparametric test. Count data were expressed as percentage or frequency and were compared using chi-squared or Fisher's exact tests. The correlation between two continuous variables was analyzed using Pearson analysis. All statistical analysis was performed with SPSS 22.0 software. A *P* value less than 0.05 was considered statistically significant.

## 3. Results

### 3.1. Baseline Characteristics

A total of 55 eyes in 50 eligible subjects (25 women and 25 men; mean age, 60.34 ± 9.76 years) with symptomatic floaters completed the 6 months follow-up and were finally included in this study. Patients reported intolerable floaters for 14.6 ± 6.2 months before resorting to YAG laser treatments. 28 (56%) patients reported that reading activities were mostly affected by floaters, 20 (40%) for driving and one half of the patients complained the bothersome floaters for all the time. Symptomatic severity of the bothersome floaters was evaluated based on the life inconvenience of patients and the IR imaging. 17 (30.91%) eyes were considered as severe, 21 (38.18%) were moderate, and the remaining 17 (30.91%) eyes were mild. Physically aging, PVD, and high myopia were significant causes of floaters. The preoperative intraocular pressure was 15.71 ± 2.4 mmHg, and all study eyes had normal IOP (Table 1).

### 3.2. YAG Treatment Safety

The 55 eyes treated with YAG laser vitreolysis received a mean of 209 laser shots. The average total energy delivered to each eye was 708 mJ (range 188–1002 mJ) for YAG lasers within the mean working time of 12.3 min (10.3–16.5 min). 38 eyes received laser treatment once, and 17 eyes required another session of YAG after one week. There was no significant YAG-related intraoperative complications, such as retinal detachment, fundus hemorrhage, and other pathological changes, except mild retina hemorrhage in one eye of a participant with previous severe floaters and PVD. The patient was given glucocorticoid, ocular nerve nutrient, and vitamin C for 2 weeks and had subsequently resolution. The follow-up mean IOP was 15.34 ± 3.27 mmHg, and there were no significant changes compared with that of baseline (*P*=0.23). Besides, there were also no significant changes in the visual acuity in the follow-up.

### 3.3. Subjective Visual Symptom Improvement

**Figure 1:**
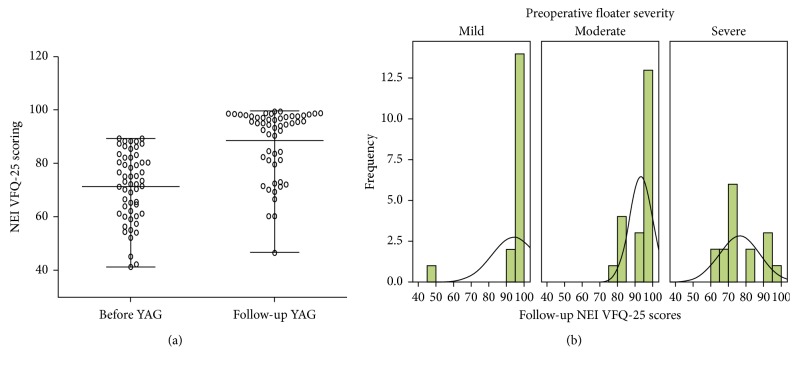
NEI VFQ-25 scores before YAG and at 6-month follow-up. (a) Changes of VFQ-25 scores before YAG and at 6-month follow-up. (b) Follow-up VFQ-25 scores in eyes with different preoperative severity.

The NEI VFQ-25 questionnaire was completed in each patient before and 6 months after the YAG procedure. The mean overall NEI VFQ-25 score was 71.44 ± 12.77, which increased to 88.54 ± 12.74 at 6 months of follow-up (*t*=11.82, *P*=0.001) (Figure 1(a)). The patients reported significantly better general and peripheral vision and fewer role difficulties and dependency on others at 6 months compared with the baseline. 4 eyes had treatment failure with 1 reporting worse symptoms and 3 reporting few improvements. 20 and 23 eyes reported partial and significant improvement in the follow-up, respectively. 8 eyes had complete resolution of the floaters in the follow-up. We evaluated the follow-up VFQ-25 score in eyes with preoperative mild to severe floaters. As shown in Figure 1(b), eyes with preoperative mild and moderate floaters had high frequency of floater resolution and significant improvement, while eyes with preoperative severe floaters were predisposed to treatment failure or partial alleviation. Besides, as shown in Table 2, there was significant difference in the clinical efficacy of YAG laser treatment between the three groups categorized by the preoperative floater severity (*P*=0.007).

### 3.4. Fundus IR Imaging Quantification of Floater Shadow Areas

**Figure 2:**
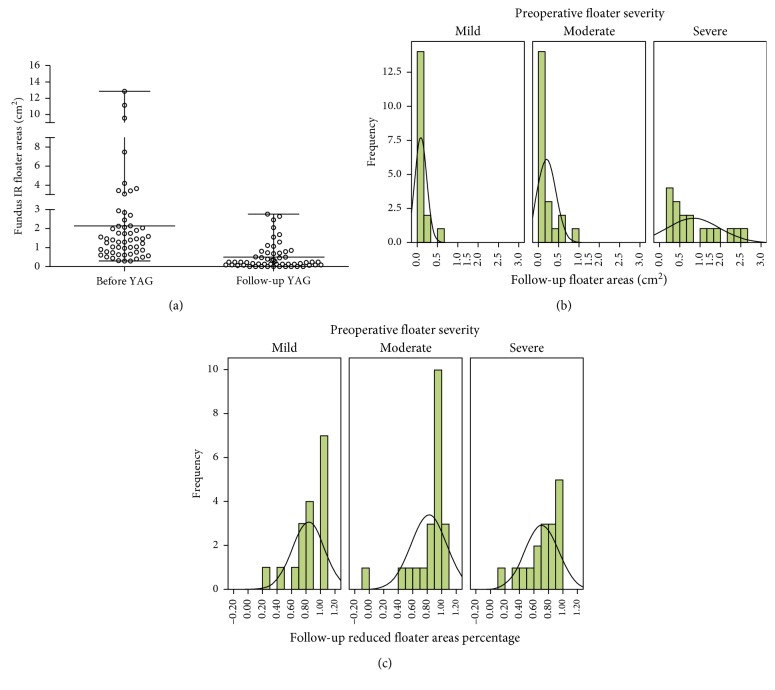
Floater shadow areas in fundus IR images before YAG and at 6-month follow-up. (a) Changes of floater shadow areas in fundus IR images. (b) Floater shadow areas in eyes with different preoperative severities. (c) Decrease percentage of floater shadow areas in eyes with different preoperative severities.

The floater shadow area in fundus IR images before YAG and at 6 months follow-up was determined using Image J software. The median of shadow areas of floaters before operation was 1.41 (0.29–12.85) cm^2^, and it decreased to 0.12 (0–2.77) cm^2^ after the operations (*t*=5.849, *P*=0.001). As shown in Figure 2(b), after classifying with preoperative severity, the residual floater shadows in mild group primarily were controlled within 0.69 cm^2^, and in moderate group, they were less than 1.38 cm^2^. However, in severe group, the residual areas had wide span reaching a maximum of 2.77 cm^2^. Likewise, the changes of floater shadow areas in the three groups also showed similar pattern (Figure 2(c)). There was significant difference in the objective clinical efficacy of YAG laser treatment between the three groups categorized by the preoperative floater severity (*P*=0.038) (Table 3).

### 3.5. Association between VFQ-25 Scores and Floater Areas

**Figure 3:**
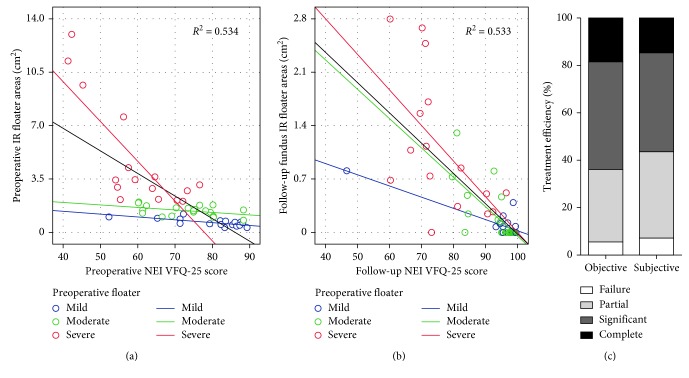
Association between objective and subjective clinical efficacy of YAG laser treatment. Scatter plot of NEI VFQ-25 questionnaire scores and floater shadow areas before YAG laser treatment (a) and at follow-up (b). (c) Comparison between objective and subjective clinical efficacy of YAG laser treatment.

To identify the consistency between subjective and objective data, we analyzed the correlation between VFQ-25 scores and floater areas using Pearson correlation analysis. It was showed that the shadow areas of floaters were negatively correlated with VFQ-25 scores before (*r*=−0.73, *P*=0.001) and after (*r*=−0.72, *P*=0.001) operations (Figures 3(a) and 3(b)). In addition, there was no significant difference in the objective and subjective clinical efficacy (*P*=0.877) (Figure 3(c)).

## 4. Discussion

Vitreous floater is a physical phenomenon occurring with ages, but it can be accelerated by various pathological factors, including ocular inflammation, hemorrhage, diabetes, or myopia. The clinical severe floaters significantly affect people's visions and quality of life. Our study demonstrated that Nd:YAG is beneficial for the elimination of bothersome floaters either by objective or subjective evaluation.

The frequency and influence of the floaters may always be underestimated. Webb et al. [10] performed a smartphone survey on the prevalence of floaters in a community sample of 603 individuals, and their data showed that 76% of the responders reported that they see floaters and 33% reported that floaters caused noticeable impairment in vision. Kim et al. [11] reported that patients with bothersome floaters always had a substantial level of psychological distress, such as depression, perceived stress, and state and trait anxiety, depending on the severity of floater symptoms. Therefore, seeking a safe and noninvasive treatment should be an option for coping with floaters [12, 13].

As a potent noninvasive treatment for floaters, Nd:YAG laser vitreolysis is considered as a safe procedure with minimal complications. It has been most frequently used for treating posterior capsule opacification after cataract surgery and anterior vitreous membrane opacification. Meanwhile, this laser treatment is also expanded to other ocular diseases, including vitreous floaters [7, 14]. However, few peer-reviewed studies on the safety and efficacy of YAG vitreolysis for treating symptomatic floaters retarded the wide application of YAG vitreolysis. It was reviewed and summarized by Milston et al. [15] in 2015 that only 91 samples were included for evaluating the YAG clinical efficacy and the reported success rates were highly variable, ranging from 0% to 100%. In addition, the authors noted that the included studies were highly variable in design and treatment protocols with small sample size, and no standardized questionnaires or objective index were used to assess the clinical efficacy. More recently, a compelling randomized clinical trial was performed to compare YAG vitreolysis with Sham YAG vitreolysis, and the results showed that 53% of the patients who underwent YAG laser treatment had completely or significantly improved symptoms, while none of the patients had resolution in the Sham YAG group [16]. In our study, we used the NEI VFQ-25 questionnaire and self-reported improvement to assess the subjective outcome. The floater shadow areas on fundus IR images measured using Image J software served as objective outcome of clinical efficacy. 63.64% of them had objective significant or complete success, and 56.37% eyes had subjective success. The success rate is higher than that in the previous study; we considered that the included patients with mild symptoms may affect the efficacy.

Due to the lack of subjective evaluation in assessing clinical efficacy of YAG, the researchers are endeavoring to use ultrasonography, infrared (IR) imaging, and optical coherence tomography (OCT) techniques to quantify the floater size and appearance. Shaimova et al. [17] believed that quantitative assessment of shadows of floaters in retinal layers is promising for clinical monitoring and optimization of treatment indications. Infrared (IR) imaging with a confocal scanning laser ophthalmoscope (cSLO) is another form of conventional fundus photography. Vandorselaer used SLO to objectively observe the position, the size, and the motility of the vitreous floaters, and he considered that floaters can be precisely located with the SLO [18]. Also, their study convinced that, with this technique, it was possible to define more precisely some eligibility criteria for Nd-YAG and to distinguish the ill-suspended from well-suspended floaters in the vitreous body. In the current study, the floater areas in IR imaging were evaluated by quantifying the grey areas using Image J. We also found that the calculated areas were negatively related to VFQ-25 scores, with a correlation coefficient reaching 0.7, indicating that it could reflect the severity of floaters.

Furthermore, the occurrence of clinical complication following laser vitreolysis is another concern in addition to the clinical efficacy in spite of few reports about these complications. The documented complications in a few small case series and individual case reports include transient elevation of IOP, focal posterior capsular opacities, refractory glaucoma, minor retinal hemorrhage, retinal breaks with detachment, cystoid macular edema, and so on. Recent studies by Noristani et al. [19], Koo et al. [20], and Sun et al. [6], respectively, described cataract, especially posterior capsular cataract with loss of integrity as a potential complication of the treated eye. It was analyzed that an inadvertent delivery of the laser power anterior to the designated target in the vitreous and the inadequate distance of the focus from the crystalline lens were responsible for the development of cataracts. Hahn et al. [21] summarized 15 complications following YAG treatment and found that focal cataracts (5 cases) and prolonged elevation of the IOP (5 cases) were major form of complications, followed by retinal injuries after YAG procedures. In our study, laser injury-related retinal hemorrhage occurred in one participant with previous severe floaters and PVD. We considered that the inadequate distance of the focus from the retina and inappropriate delivery of energy might be the potential causes of retinal injury. In this case, glucocorticoid was given to suppress inflammation and improve microcirculation, ocular nerve nutrient to promote lesion recovery, and vitamin C to reduce oxidative stress.

There are inherent limitations to this retrospective analysis lacking a randomized control arm. Our study preliminarily concluded that the clinical efficacy of YAG laser treatment was achieved depending on the baseline severity of the floaters, and the result should be evaluated in more clinical practice. Besides, the clinical efficacy and indications or contraindications of YAG should be further assessed in stratified populations with various pathological causes or baseline characteristics. Meanwhile, the quantitative IR imaging technique in this study has its own limitations. Its sensitivity depends on the contrast between floaters and background, and circling the target areas can be influenced by the weak boarding. In addition, since the floater is sometimes irregular in appearance and stereoscopically scattered, the casted shadow could not completely reflect the actually existed floaters.

Above all, we evaluated the clinical efficacy of Nd:YAG laser in treating symptomatic floaters and intended to define the objective and subjective outcome at 6 months after the procedure. The data showed that significant and complete resolution of vitreous floaters was achieved in 63.64% of the studied eyes as determined by objective quantification and in 56.37% eyes by self-reported data. Nd:YAG is beneficial for the treatment of bothersome floaters, especially for those with mild or moderate floaters. IR fundus photography-based quantification of floaters may be a promising tool in assessing the objective outcome of YAG treatment.

## Figures and Tables

**Table 1:** Baseline characteristics of the included patients.

Variables	Means ± SD/*n* (%)
Age	60.34 ± 9.76
Female	25 (50%)
BMI (kg/m^2^)	20.26 ± 4.15
Time from onset (months)	14.6 ± 6.2
Unilateral/bilateral	50/5
Activity most affected by floaters	
Reading	28 (56%)
Driving	20 (40%)
All of the time	25 (50%)
Baseline intraocular pressure (mmHg)	15.71 ± 2.4
Baseline visual acuity (logMAR)	0.28 ± 0.18
Preoperative symptomatic severity	
Mild	17 (30.91%)
Moderate	21 (38.18%)
Severe	17 (30.91%)
Cause	
Physically aging	15 (27.27%)
Posterior vitreous detachment	21 (38.18%)
Liquified degeneration of vitreous body	6 (10.91%)
High myopia	13 (23.63%)

**Table 2:** Follow-up self-reported improvement by patients.

Preoperative severity	Follow-up subjective improvement				Statistics
	Failure	Partial success	Significant success	Complete success	
Mild (*n*=17)	1	3	6	6	*P*=0.007
Moderate (*n*=21)	0	5	10	2	
Severe (*n*=17)	3	12	7	0	
Sum	4	20	23	8	

**Table 3:** Follow-up improvement evaluating by floater shadow areas in fundus IR images.

Preoperative severity	Follow-up objective improvement				Statistics
	Failure	Partial success	Significant success	Complete success	
Mild (*n*=17)	1	2	7	7	*P*=0.038
Moderate (*n*=21)	1	6	11	3	
Severe (*n*=17)	1	9	7	0	
Sum	3	17	25	10	

## Data Availability

The data used to support the findings of this study are available from the corresponding author upon request.
